# Canopies, the Final Frog-tier: exploring responses of a specialist treefrog to prescribed fire in a pyrogenic ecosystem

**DOI:** 10.1186/s42408-022-00148-1

**Published:** 2022-10-22

**Authors:** Ian N. Biazzo, Pedro F. Quintana-Ascencio

**Affiliations:** Department of Biology, University of Central Florida, 4000 Central Florida Boulevard, Orlando, FL 32816 USA

**Keywords:** Amphibians, *Dryophytes femoralis*, Pinewoods treefrog, Florida, Mark-recapture, Pine flatwoods, Canopy, Fire effects, Bayesian, Single rope technique

## Abstract

**Background:**

Pine flatwoods of the southeastern United States were shaped by frequent fires. Land managers use prescribed fires to control fuels but also to restore historical fire dynamics. Broad outcomes of this practice are well-understood, but impacts on many organisms are still being explored. Frogs, for example, have upland and wetland requirements, limited mobility, and skin susceptible to desiccation. Treefrogs spend most of their lives in uplands away from water. When fire approaches, animals may escape to an unburned area, shelter in place, or be killed by the fire. We examined which of these mechanisms is the prevailing short-term response for a specialist treefrog in a pyrogenic flatwood system.

**Results:**

We assessed the short-term impacts of prescribed fire on the dynamics of an upland flatwood specialist, the pinewoods treefrog *Dryophytes femoralis*, using a replicated before-after-control-impact field experiment. We set pipes as treefrog refugia at 3 m, 6 m, 9 m, and 9^+^ m in 12 pine trees spread evenly across two treatments: reference trees in units burned in 2020 and trees in units with 2021 prescribed fire. Prescribed fires occurred on 16 April and 21 July 2021. Every 2 weeks between 5 March and 5 September, we checked pipes for frogs and assigned them unique color marks. We observed 78 individuals with 199 additional recaptures. We modeled abundance (as raw counts), survival, and vertical movement using mark-recapture methods, multi-state, and mixed linear models with a Bayesian framework. Survival and recapture were comparable among prescribed fire treatments, but abundances and movement probability varied. Frogs in trees in areas burned during the study were more likely to stay in place and less likely to descend to lower heights. We observed more frogs in trees after a 2021 fire compared to reference trees.

**Conclusions:**

The prevailing mechanism for resiliency to fire for pinewoods treefrogs was migration up large pines, then likely recolonization to lower vegetation layers when plants regreen post-fire. This substantiates conclusions from other works that the integrity of mature pines is key to sustaining native biodiversity. Future work and management should consider the three-dimensional structure of habitat when developing burn prescriptions and study designs.

## Resumen

**Antecedentes:**

Las planicies de pinos del sudeste de los EEUU fueron modeladas por fuegos frecuentes. Allí, los manejadores de tierras usan las quemas prescriptas para controlar los combustibles vegetales, pero también para restaurar la dinámica de los incendios causados por rayos. Los resultados a gran escala están bien comprendidos, aunque los impactos sobre muchos organismos son todavía objeto de estudio. Las ranas, por ejemplo, tienen requerimientos particulares tanto en humedales como en tierras altas, dada su limitada movilidad y su piel sensible a la desecación. Las ranas que habitan en árboles (*treefrogs*) pasan la mayoría de sus vidas en tierras altas alejadas del agua. Cuando un incendio se aproxima, los animales pueden escapar hacia un área no quemada, guarecerse del fuego mediante una cobertura protectora, o ser muertas por el fuego. Examinamos cuál de esos mecanismos es la respuesta dinámica más prevalente en el corto plazo para la persistencia de una rana de árbol especialista en ese ecosistema pirogénico de planicies de pinos.

**Resultados:**

Determinamos los impactos a corto plazo de una quema prescripta en la dinámica de un especialista de esas planicies, la rana de los pinos *Dryophytes femoralis,* usando un experimento de campo replicado de antes-después- y control de impacto. Usamos tubos como refugios de ranas a 3, 6, 9, y más de 9 metros en 12 pinos distribuidos uniformemente entre dos tratamientos: árboles de referencia en unidades quemadas en 2020 y árboles en unidades quemadas en 2021 mediante quemas prescriptas. Las quemas prescriptas fueron realizadas en 16 de abril y 21 de julio de 2021. Cada dos semanas entre el 5 de marzo y el 5 de setiembre, revisamos los tubos con ranas y le asignamos marcas de color únicas. Observamos 78 individuos con 199 recapturas adicionales. Modelamos la abundancia (como conteos crudos), supervivencia y movimientos verticales usando el método de marcas de recaptura, estados múltiples y modelos lineales mixtos dentro de un marco Bayesiano. La supervivencia y recaptura fueron comparables entre los tratamientos de quemas prescriptas, aunque la abundancia y la probabilidad del movimiento variaron. Las ranas en los árboles en las áreas quemadas durante el estudio fue más probable que se quedaran en su lugar a que descendieran a lugares más bajos. Observamos más ranas en árboles luego de la quema del 2021 comparadas con las que estaban en árboles de referencia.

**Conclusiones:**

El mecanismo prevalente para la resiliencia al fuego para las ranas de árboles fue la migración hacia pinos más grandes, y luego recolonizar lugares más bajos cuando las planteas reverdecen en el post-fuego. Estas conclusiones se consustancian con otros trabajos en que la integridad de los pinos maduros es clave para sostener la biodiversidad nativa. Trabajos de investigación futuros y prácticas de manejo deberían considerar la estructura tridimensional del hábitat cuando se desarrollen prescripciones de quema y diseños de estudio*.*

## Background

Fire, a common global disturbance, may have an immediate impact on organisms via mortality or emigration, but also have longer-lasting impacts due to habitat alteration, resource availability, and chemical leaching (Wilbur and Christensen 1983; Russell et al. 1999; Noss and Rothermel 2015; Jones et al. 2020). In some regions and ecosystems, this natural disturbance is frequent enough that it becomes a major driver of the population dynamics for many species (Kauffman 2004; Noss 2013). Pine savannas of the southeastern US are closely linked to fire. Historically, these ecosystems experienced relatively frequent fires (1–5 years) that were lightning-induced or initiated by Native Americans. These fires maintained structural and compositional properties of the ecosystems, affected their chemical attributes, and influenced the propagation of future fires (Komarek 1968; Barnett 1999; Huffman 2006; Noss 2013). Many of the constituent plant species in these fire-prone ecosystems require frequent fire for reproduction, and most recover quickly from burns (Outcalt 2000; Florida Natural Areas Inventory (FNAI) 2010; Noss 2013 and those cited within).

In the United States (US), prescribed fire is one of the most common methods used by land managers to attempt to restore this critical ecological process while safely reducing fuel loads and burn intensity on natural lands (U.S.D.A., U.S.D.I 2002; Ryan et al. 2013). While optimal fire intervals for ecosystem persistence are relatively well understood, the potential for fine-scale modifications to accommodate species responses to fire is an important consideration for scientists and land stewards (Noss 1987, 1996). Fine-scale adjustments within coarse-filter conservation and management plans have been effectively implemented for many species, such as pre-burn understory thinning for cavity trees of red-cockaded woodpeckers *Dryobates borealis* Vieillot (Williams et al. 2006), deliberate hardwood patch protection for southern fox squirrels *Sciurus niger niger* (Perkins et al. 2008), and vehicle buffers to prevent burrow collapse for gopher tortoises *Gopherus polyphemus* Daudin (Smith et al. 2015).

Prescribed fire management is mainly based on outcomes for plants (Driscoll et al. 2010) while recognizing the indirect impacts on animal populations. Animals may also respond directly to a fire, or any other disturbance, and outcomes can be generalized to dispersing, sheltering in place, or dying (e.g., Peterman et al. 2011). While these mechanisms occur simultaneously at the individual level, each can have different population impacts. Populations going through a mass mortality or significant exodus event may experience a temporary or permanent extirpation (e.g., Morris et al. 2011). If the prevailing mechanism is to shelter in-place, however, a local population may be present immediately post-disturbance. Studies that focus on individual- and population-level mechanisms, and different spatiotemporal scales, are therefore necessary to fully understand fire-influenced dynamics (Odum et al. 1979; Pickett and White 1985; Russell et al. 1999; Driscoll et al. 2010; O'Donnell et al. 2016).

Amphibians are typically not considered in pyrogenic systems, yet they are good models for looking at prescribed fire impacts. Amphibians have relatively limited mobility, are sensitive to chemical perturbations, require special microhabitats, and have been experiencing enigmatic declines worldwide (Blaustein et al. 1994; Stuart et al. 2004). An overwhelming majority of amphibian studies to date have focused on collecting data at breeding events in wetlands, which often represents only a snapshot of this group’s life histories (Boughton et al. 2000; Pilliod et al. 2003; Klaus and Noss 2016; Robertson et al. 2018). Treefrogs, in the family Hylidae, are particularly interesting because many require both upland and wetland ecosystems for their life cycle but spend most of their lives in those uplands where they readily ascend into tree canopies. Studies and anecdotal observations note that at least some age classes of treefrogs in the Southeast prefer to be above the ground (Wright and Wright 1949; Boughton et al. 2000; Windes 2010; I.N. Biazzo personal observations). To the best of our knowledge, no studies have looked at hylid occupancy above 4 m in height in the US (e.g., above the understory stratum in pine-dominated systems) and considered impacts of fire or other disturbances on their vital rates and other population attributes.

**Fig. 1:**
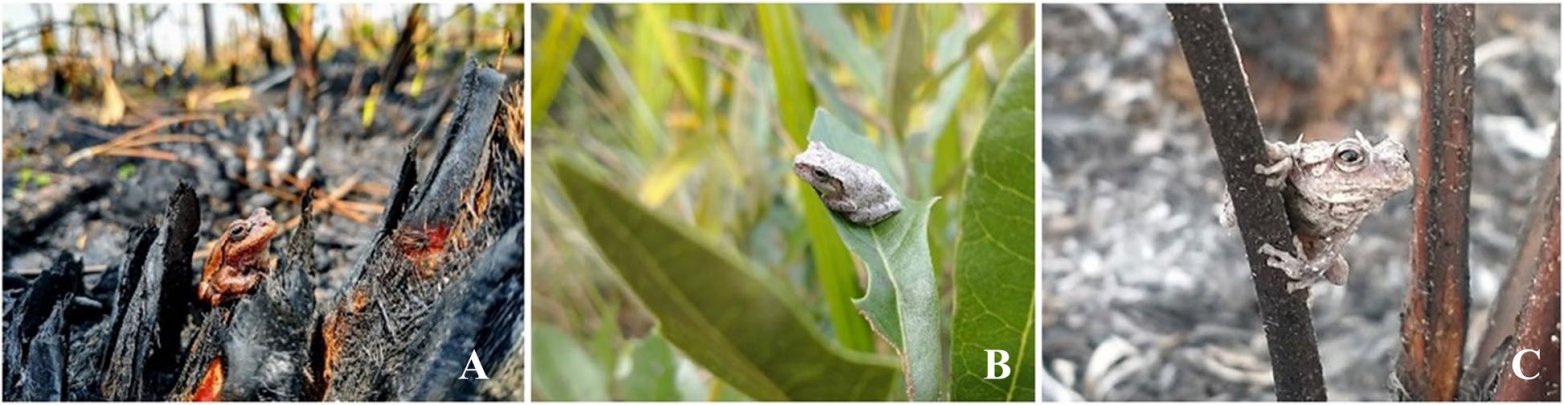
Pinewoods treefrogs. **A** Adult in burned area 1 day after a prescribed fire. **B** Juvenile in moisture conserving position. **C** Adult covered in ash debris hours after a prescribed fire. Photo credits: Ian Biazzo

We used a before-after-control-impact experimental design combined with mark-recapture to examine the impacts of prescribed fire on apparent survival, movement, and abundance (i.e., raw counts) of treefrogs in pine flatwoods in central Florida, US. To observe treefrogs, which use natural cavities to avoid desiccation and predation, we set polyvinyl chloride (PVC) pipes in trees as artificial refugia which they could enter and exit at will (Buchanan 1988; Boughton et al. 2000; Schurbon and Fauth 2003; Zacharow et al. 2003; Glorioso and Waddle 2014). While the pine flatwoods are home to four native treefrog species in central Florida, 99% of individuals observed in our study were one specialist species, the pinewoods treefrog (*Dryophytes (Hyla) femoralis* Bosc, Fig. 1) (Klaus and Noss 2016). We focused on the following questions regarding this specialist species: (1) What are the base-level abundances, apparent survival estimates, and movement estimates of frogs in trees before a prescribed fire? (2) Does a prescribed fire cause short-term changes in these parameters? (3) If so, then which is the prevailing mechanism of short-term population change after a prescribed fire?

## Methods

### Study site

**Fig. 2:**
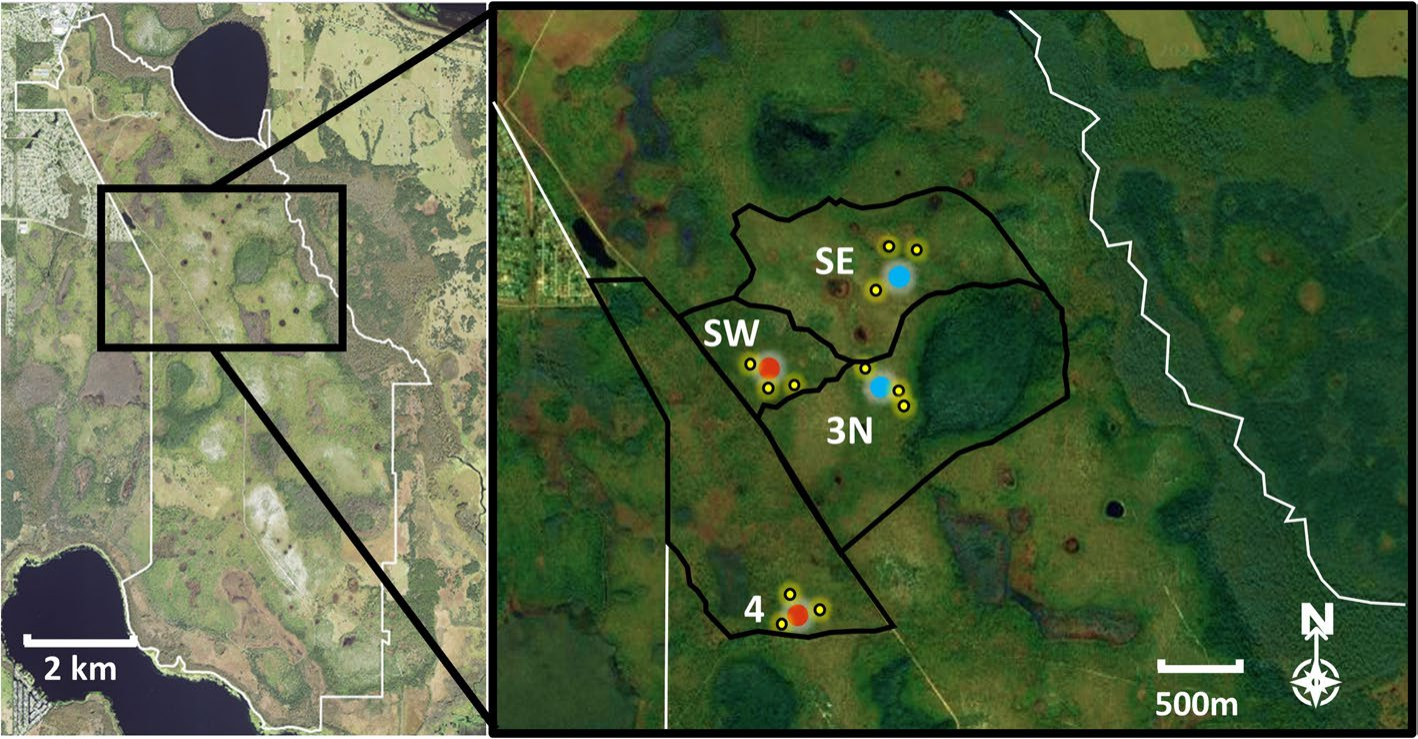
Left: Map of The Nature Conservancy’s Disney Wilderness Preserve, Poinciana, FL. Right: Zoomed in map of TNC-established burn units and trees chosen for climbing. Blue dots are marshes in units burned in 2020 and red dots are marshes in units with prescribed fire in 2021 during the study. Yellow dots mark the layout of the 12 pine trees used in the study

We focused our study on pine flatwood ecosystems around depression marshes in Disney Wilderness Preserve (DWP) near Poinciana, FL, US (28.129876°, −81.429310°, Fig. 2). DWP is a 4654-ha preserve owned and managed by The Nature Conservancy (TNC) and is part of the Greater Everglades watershed. The preserve is mainly composed of pine flatwoods with interspersed swamps, freshwater marshes, hammocks, and scrub. In 1992, the property, then a cattle ranch, was purchased as a restoration mitigation site by the Walt Disney World Corporation and then later transitioned to TNC. Ecological and hydrological restoration, which included filling in a heavily ditched landscape and improving the hydroperiod, occurred from 1994 to 2012 and was deemed successful in 2012. Current management includes growing season (March–July) prescription burns at ~3-year intervals. Temperatures recorded by an on-site National Ecological Observatory Network (NEON) station ranged from 8 to 35 °C through the study (March–September), and monthly precipitation was 4.5, 111.4, 16.6, 230.3, 207.7, 125.9, and 104.4 mm, respectively (National Ecological Observatory Network, 2022a, b).

### Experimental design

We selected 12 longleaf pine trees (*Pinus palustris* Mill.) as replicates spread across four different fire management units with different burn schedules. We chose living trees with a minimum height of 9 m, a safe anchoring branch in the crown (for rope), no obvious lightning or rotting damage, and at least 25 m from any fire breaks. If more than three trees met these criteria within the unit, then we randomly chose three. Spreading the trees across multiple units increased our chances of having at least one prescribed fire (this was a COVID-19 pandemic year and burns were frequently canceled). Two units, Unit Scrub West (SW) and Unit 4 (4), were previously burned in 2017 and scheduled to burn in 2021 during our study while the other units, Unit Scrub East (SE) and Unit 3N (3N), burned a year before the study in 2020. Trees in these 1-year time-since-fire (TSF) units served as experimental field references to control for short-term post-fire responses (Fig. 2). For convenience, any use of “pre-fire” and “post-fire” refers only to the 2021 prescribed fire conditions. The burn crews at DWP conducted prescribed burns on the experimental fire Unit 4 on 16 April 2021 and Unit Scrub West on 21 July 2021.

**Fig. 3:**
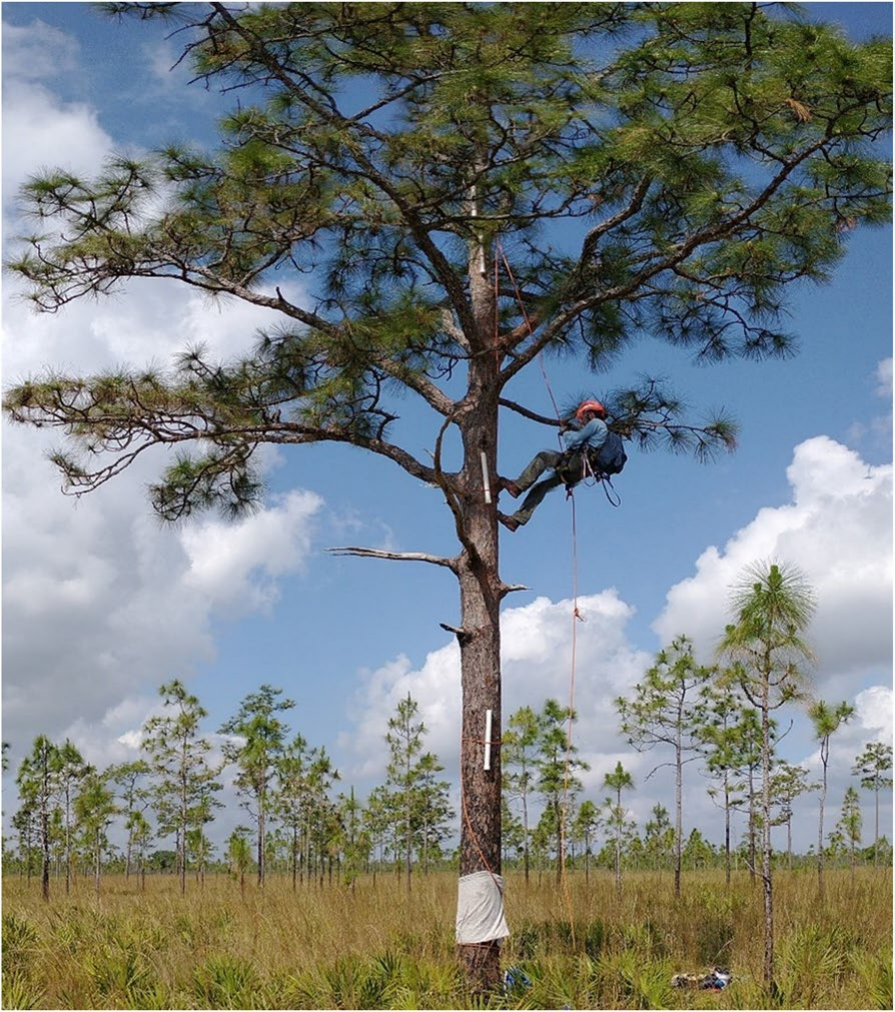
Tree setup and data collection. We set white PVC pipes at 3 m, 6 m, 9 m, and 9^+^ m in each tree and checked them using the single rope technique and climbing equipment. The last height represents the highest pipe that could be placed and varied between 9.5 and 12 m. Photo credit: Rachel Gutner

Between 15 and 21 February 2021, we attached PVC pipes as treefrog refugia in the 12 chosen pine trees (Buchanan 1988; Boughton et al. 2000; Schurbon and Fauth 2003; Zacharow et al. 2003; Bartareau 2004; Glorioso and Waddle 2014). All pipes were 61 cm long, 3.8 cm internal diameter, 330 psi, white, bottom-capped, fitted with a drainage hole at 10 cm from the bottom and a screw hole at the top, and secured to the tree with a drill and bungee so they faced inward towards the marsh. We set pipes at approximately 3 m, 6 m, 9 m, and, when tall enough, 9^+^ m (Fig. 3) using a single rope technique (SRT) with rapid ascension and descension equipment (RAD). This breakdown was chosen based on two previous Florida studies that found no significant difference in PVC success between 2 and 4 m from the ground (Boughton et al. 2000; Windes 2010). The final height, 9^+^ m, varied per tree based on the tallest safe attachable height, which ranged from 9.5 to 12 m. This resulted in 3–4 pipes per tree, and 45 total PVC pipes across the 12 pines. We draped paracord over the anchor branches to minimize subsequent rope setup time.

### Data collection

We started sampling on 5 March 2021, approximately 2 weeks after the setup. This latency period allowed frogs time to discover the pipe refugia and is consistent with similar studies (Boughton et al. 2000, Zacharow et al. 2003; Myers et al. 2007; Windes 2010). We continued sampling every 2 weeks until 5 September 2021, near the peak of Florida hurricane season, which resulted in 14 sampling events. One person ascended each tree using the SRT-RAD setup, inspected pipes for treefrogs, carefully emptied frogs into large transparent plastic bags, safely secured the bags in their backpack, emptied the pipe of debris and water, replaced the pipe, and descended the tree with the frog(s). Once on the ground, we measured snout-vent length (SVL) and marked individuals with a unique combination of visible implant elastomer markers (VIE, Northwest Marine Technology, Olympia, WA, US). We marked frogs on 1–4 locations ventrally: left or right inner thigh or inner calf. If a frog was a recapture, then we recorded its color combination and no further marks were given. All processing was done through the plastic bag. We released frogs at the base of the study tree after the climbing equipment was disassembled rather than the height of capture for consistency, to reduce biasing future recaptures, and to decrease climbing and handling times. Total handling time was typically only a few minutes.

We fully depended on PVC pipes for treefrog data collection. While effective and commonly used, there are two main concerns. First, these are not natural elements of the landscape and therefore introduce artificial biases that could disrupt background behaviors and population dynamics. Second, and relatedly, this sampling design may not be representative of the true population. Specifically, the use of PVC pipes has not been validated as a random and unbiased way to sample a population of frogs. We mitigated these concerns by making sure we used the pipes consistently across all trees and treatments, making any artificial impacts and biases shared throughout.

To establish basic comparisons of our trees, we combined the physical environmental data for each tree and used them to make averages for each unit. We recorded tree height, diameter at breast height (DBH), distance to nearest wetland, number of branches >30.5 cm (12 inches) off main trunk, and percent crown height. We measured neighboring tree density 5 m, 10 m, and 15 m from the study tree.

### Generalized linear mixed modeling

We used generalized linear mixed models with a Bayesian framework to examine short-term prescribed fire impacts on treefrog abundance (counts of new and counts of total individuals). We used a Poisson distribution to model frog counts and applied a log link function to represent spatiotemporal variation as a linear model (Eqs. 1, 2, 3, and 4). Individual counts in this approach underestimate true population sizes, but it captures the population trend over time when the observation process is consistent across the study (Kéry and Schaub 2012 and those cited within). We tested hypotheses based on combinations of tree location (*δ*_*j*_), sampling time effects (*ɣ*_*i*_), and the three 2021 prescribed fire conditions (*β*_*p*_): no fire or pre-prescribed fire, 0–6 weeks post-fire (available for 6 trees), and 6^+^ weeks post-fire (available for 3 trees). We chose a 6-week cutoff for three reasons: (1) the ecosystem regreens quickly after a prescribed fire, (2) the data showed this is enough time to detect a short-term signal, and (3) it provided a way to look at possible shifts back to pre-prescribed fire conditions. Recognizing the potential for environmental differences among trees, and variation due to sampling time, we modeled tree location and time as normally distributed random effects (Eqs. 3 and 4). Trees were not identical across the study, but we are convinced they were interchangeable representatives of the larger population of longleaf pines.

We used a widely applicable information criterion (WAIC; Gelman et al. 2014) score, which is an appropriate criterion for Bayesian applications, to evaluate support for each model and then compared the posterior probabilities of the most supported model to test for impacts of the 2021 prescribed fires. Bayesian models are well suited to evaluate our study response, short-term prescribed fire impact, because they emphasize the distribution of the parameter values and of the model predictions, rather than solely a mean with error. This is particularly important when the distribution of the outcomes is not normally distributed, since the mean would not be a good descriptor of the response. The resulting probability distributions convey critical information that allow us to better evaluate, and visualize, the uncertainty of our predictions and probabilities of different outcomes by comparing the shape of the curves and overlaps between them (Quintana-Ascencio et al. 2022).

1 $${\mathrm{Counts}}_{i,j}\sim \mathrm{Poisson}\ \left({\lambda}_{i,j}\right)$$

2

3 $${\delta}_j\sim \mathrm{Normal}\ \left(0,{\sigma_{\delta}}^2\right)$$

4 $$\gamma_j\sim\mathrm{Normal}\;\left(0,\sigma_\gamma^2\right)$$

### Multi-state modeling

From the capture and recapture data, we created a matrix of capture histories showing presence at a specific height and absences for each frog. To evaluate the vertical transitions, we used a categorical multi-state approach where the “states” were the different height levels (3 m, 6 m, 9 m, and 9^+^ m) in the trees along with a fifth state representing death. Stochastic ecological processes govern the transitions between each height, but dead individuals deterministically remain dead. Equation 5 shows how the height-specific survival (*ϕ*_*i*_) and movement (*α*_*i*, *j*_) probabilities are combined to form transition estimates. For example, for an individual to transition from 3 m at time *t* to 6 m at *t*+1 means that it survives with probability *ϕ*_3_ and moves with probability *α*_3, 6_. While we cannot observe all these transitions every time, height-specific recapture probabilities (*p*_*i*_, *p*_*i*_ < 1) can be estimated from consecutive ratios of observed and unobserved individuals from time *t* to *t*+Δ*t*. An observation matrix (Eq. 6) of these recapture probabilities links the true state to the observed state using likelihood drawn from a categorical distribution. The observations (columns) are conditional on the true states (rows) for each individual and are also governed by stochastic processes (except for dead individuals which remain unobservable). For example, a frog at 3 m has a non-zero recapture probability at only 3 m. The model uses all the capture histories to produce survival and recapture estimates for each state, or height, and transition probabilities for each possible movement up or down from time *t* to time *t*+1. Each row in Eqs. 5 and 6 assumes the total possible outcomes for a given height, so each transition is constrained on the probability scale [0,1] and the rows each sum to 1. Temporary emigration out of pipes or trees may lower estimates of recapture, but if it is non-Markovian, then it is considered random and does not bias estimates of apparent survival probabilities.


5


6

We ran separate models for trees with fire in 2021 during our study and reference trees burned in the previous year, then we compared the posterior probabilities. To facilitate computation, we incorporated vague, weakly informative Bayesian priors for survival based on estimates from work on a congeneric in central Florida and based on an ongoing study in this same system (Windes 2010; Biazzo et al., unpublished data). True survival cannot be effectively disentangled from permanent emigration in the multistate models, so we report estimates as “apparent survival” with the understanding that apparent survival typically underestimates true survival. The high site fidelity in this study suggests apparent and true survival may be similar, but further studies need to confirm this. Models were run using R version 3.4.4 (R Core Team 2019) and version 2.18.0 of Stan (Stan Development Team 2018; Carpenter et al. 2017) based on modified code for population analyses based on Kéry and Schaub 2012 and Itô 2015 (modified code available in the supplemental information).

## Results

### Environmental factors

Overall, trees chosen in each unit had similar average heights (11.8–15.2 m), percent live crown (37.5–49.2%), DBH (34.2–42.1 cm), few to no branches below 6 m, and few to no neighboring trees within a 5-m radius. There was more variation in average distance to the nearest wetland (23.3–71.7 m) and tree density within a 15-m radius (2.7–15 trees, Table 1).

**Table 1:** Summary of physical environmental data for reference trees (Units SE, 3N) and trees in areas with prescribed fire in 2021 (Units 4, SW). Charted numbers are means based on the three study trees in each unit. Branches columns indicate the average number of branches between the pipes. Crown is measured from the lowest living branch and higher. Wetland distance is based on the distance to the nearest ephemeral marsh. The nearest neighbor is the distance to the single closest living tree >2 m tall. Trees within 5, 10, and 15 m were all counted if >2 m tall. The mean diameter at breast height (DBH) at 1.4 m is based on all neighbors counted within 15 m. Finally, the DBH of the study trees was measured at 1.4 m

**Environmental comparisons**							
	Height (m)	Branches ground–3 m	Branches 3–6 m	Branches 6–9 m	Branches 9–9^+^ m	Branches >9^+^ m	Crown%
Unit SE	13.50	0.33	3.00	6.67	2.50	5.67	37.50
Unit 3N	11.83	0.00	2.33	8.00	3.00	5.00	47.50
Unit 4	15.17	0.00	1.00	4.67	5.33	4.33	45.00
Unit SW	14.17	0.00	1.00	5.00	5.00	4.67	49.17
	Wetland distance(m)	Nearest neighbor (m)	Trees within 5 m	Trees within 10 m	Trees within 15 m	Mean DBH of neighbors	DBH (cm)
Unit SE	64.00	2.83	1.67	7.00	15.00	12.45	36.30
Unit 3N	71.67	15.00	0.00	0.00	2.67	16.53	37.93
Unit 4	23.33	6.60	1.00	8.33	7.33	10.33	42.13
Unit SW	27.67	6.83	0.33	3.67	14.33	8.49	34.17

### Mark-recapture

We observed 78 pinewoods treefrogs with 199 recaptures and 2 that escaped before identification. We detected one individual congeneric, *D. squirellus* Daudin, which was not used in analyses. For the multistate analysis, we excluded only one pinewoods treefrog data point because marks on the recaptured frog had faded. Of the 199 recaptures, only one frog moved between trees (distance ~65 m) and it only moved once. Only 29 frogs (38%) were never recaptured while 48 frogs (62%) were recaptured at least once. The longest capture history was one frog with 14 captures, or all sampling events in the study. The average capture history among all individuals was 3.6 captures and among those recaptured at least once it was 5.1 captures. Finally, we observed two dead recaptured treefrogs in 9-m pipes that likely died due to convective heat or heavy smoke from the prescribed fire. They were found in the tree with the highest bark char height along with several dead green anoles, *Anolis carolinensis* Voigt, but none showed visual signs of fire damage. We observed frogs in 11 out of 12 trees and at all heights, with 57 frogs captured at 3 m, 52 at 6 m, 86 at 9 m, and 81 at 9^+^ m (Table 2).

**Table 2:**
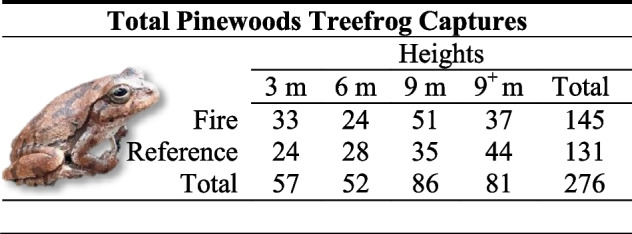
Total pinewoods treefrog occurrences (new and recaptures) per height (3 m, 6 m, 9 m, and 9^+^ m) and experiment treatment. Fire represents frogs found in trees in units that had a prescribed fire during the study, whereas Reference represents frogs in reference trees that did not burn during the study year

### Abundance

**Fig. 4:**
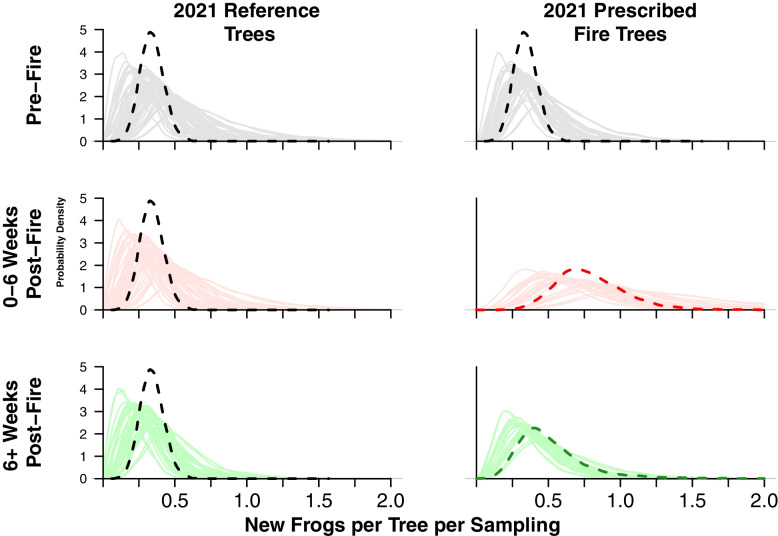
Bayesian posterior distributions for models of new frog abundance with reference trees on the left and 2021 prescribed fire trees on the right. Top: response before the 2021 fires. Middle: response during the time in each treatment 0*–*6 weeks after a prescribed fire happened. Bottom: response during the time in each treatment 6^+^ weeks following a 2021 prescribed fire. Each graph represents the model posterior prediction for the number of new frogs in trees during the time period and treatment condition. Bolded dashed lines show the posterior distribution of the average effects without fire (black), for the time period 0*–*6 weeks after a prescribed fire (red), and for the 6^+^ weeks following a prescribed fire (green). In the background are the posterior prediction for actual trees and sampling interval by fire treatment. The *X* axis presents the possible posterior estimates of the number of new frogs and their corresponding probability density is presented in the *Y* axis. The 0*–*6-week post-fire model (center) indicates a higher chance of finding more new frogs per tree in trees in areas that burned in 2021 (center right) than without fire in 2021 (center left), but both treatments are similar during the other two temporal conditions

The number of new frogs observed in trees varied throughout the study, with likely seasonal differences between spring and summer months and corresponding burns (Fig. 4). According to the WAIC comparisons, where weights relate to the predictive power of the models, the model most likely explaining abundance variation included fire as a fixed effect and both time and tree location as random effects (Table 3). This was consistent for total frog counts (i.e., new frogs and recaptured frogs, *n*=279) and for just new frogs (*n*=78). When modeling new frogs only, the weight of the null model (average, constant over space and time) was close to the model for fire impact with random effects. This is likely an artifact of the relatively small sample size of new frogs (only 78), as this similarity was not seen for total frogs (*n*=279). The model for new frogs with the best support showed a higher probability of finding new frogs 0–6 weeks post-fire, then a shift back to pre-fire or no fire conditions 6^+^ weeks after the fire (Fig. 4). We plotted the entire distribution of the resulting posterior probability densities to show they deviate from normal distributions. These also have the advantage that the area under any part of the curve is interpreted as the probability of observing that many frogs per tree per sampling time.

**Table 3:** Widely applicable information criterion (WAIC) statistics for models of abundances separated by counts of new frogs and counts of total frogs. We evaluated responses as a function of fire, time (sampling event), and tree. †Random effect. The WAIC differences (dAIC) and relative model weights are based on comparisons of each model with the most likely model

Generalized linear model results					
Model	Total frogs		Model	New frogs	
	dAIC	Weight		dAIC	Weight
Fire + time (†) + tree (†)	0.0	1.00	Fire + time (†) + tree (†)	0.0	0.57
Time (†) + tree (†)	17.78	0.00	Null (mean only)	1.33	0.29
Null (mean only)	120.67	0.00	Time (†) + tree (†)	2.80	0.14

### Multi-state models

**Fig. 5:**
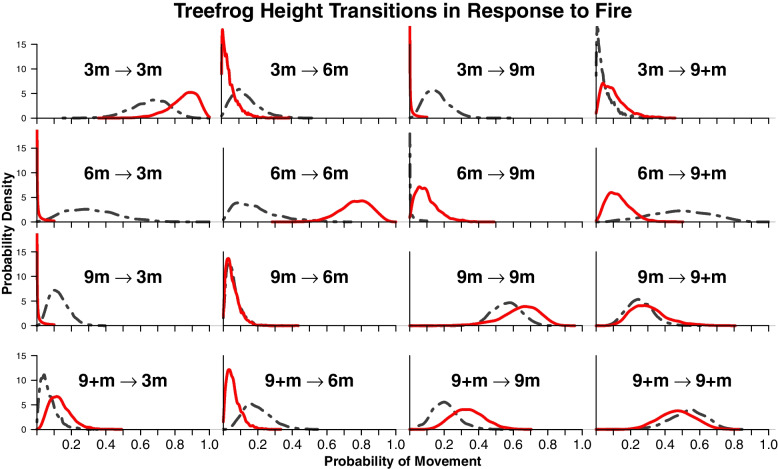
Bayesian posterior distributions representing the chances of transition between heights from the multistate model. The main diagonal is the probability of remaining at the same height between time *t* and *t+1*. The red solid line represents trees in plots with prescribed fire in 2021, and the gray dotted line shows reference trees without fire in 2021. The *X* axis shows possible posterior estimates of the chances a frog transitions between heights and the probability densities are presented in the *Y* axis

Among all heights, we estimated apparent survival between 77 and 83% in trees with 2021 prescribed fire and 62–91% in reference trees, and recapture rates of 63–85% and 62–88%, respectively. We found frogs in trees with the recent 2021 fires were overall more likely to remain at one height instead of moving up or down to other heights (Fig. 5, diagonal). Movement between the top two heights, 9 m and 9^+^ m, was similar across the study (Fig. 5, bottom right). There was a higher probability of descending to lower heights of 3 m and 6 m in trees in areas without 2021 fires. Since Bayesian posterior distributions are interpreted as probabilities, we find it is useful to see the full extent and shape of the distribution curves and any potential overlap between the two treatments.

## Discussion

We observed an increase in abundance of a specialist treefrog in trees directly after 2021 prescribed fires compared to a 1-year TSF reference, before the 2021 fires, and 6^+^ weeks postfire. These findings provide support for a refugia hypothesis, suggesting that these animals persisted within their habitat by climbing up trees rather than evacuating to unburned areas. Many frogs entered our study trees after the fire, remained for several weeks, then were gradually not seen again. The parabolic shape of this response and lack of an obvious mortality event post-fire across all treatments suggests these individuals descended back into the lower subcanopy, understory, and shrub layers once the plants regreened, which is consistent with other studies. While sampling at and below breast height in a similar system, Schurbon and Fauth (2003) observed a lower abundance of treefrogs in a <1-year TSF area compared with areas burned in the previous year. Given the high site fidelity and low death by fire observed, temporary refugia in the tall trees seem to be a dominant mechanism for this species persisting in a fire-prone landscape. This conclusion supports other studies that highlight the importance of maintaining some healthy, mature trees during burns (Williams et al. 2006).

While our data indicate a short-term increase of frogs in trees immediately after a prescribed fire, few dead frogs, high apparent survival estimates, and evidence of movement up and down in the trees, there are caveats to consider for future work. A limitation is the study length itself, as it lasted only 6 months, covering 14 sampling occasions in a single preserve. Future work should include more years of data and more sites to better disentangle any spatial or seasonal effects from fire impacts. Another limitation to consider is the impact of artificial refugia on behavior, survival, and sampling biases. For example, the PVC pipe may support different numbers of frogs or offer a different microclimate than natural shelters. Despite these limitations, it is essential to follow the experimental model presented here to expand on typical time-since-fire monitoring, which often lacks structured data before the prescribed fire for animals. The already complex combinations of fire effects, which can be direct and indirect but also vary spatiotemporally (Russell et al. 1999), and subsequent management decisions could intensify as ecosystems buffer other anthropomorphic threats. The taxonomic groups and habitats that are vulnerable today may not be the same ones that are at risk in the future.

The tree bole itself is both habitat and linear conduit between the ground and canopy strata for non-volant animals. As habitat per se, boles are spatially isolated and the interactions of trunk-specialist species are understudied compared to those at canopy and ground layers (Menzel et al. 2004; O’Hanlon 2011). Similarly, the way animals use boles as highways or temporary habitat between the ground and canopy is often overlooked (e.g., Proctor et al. 2002). On several occasions, we observed treefrogs upon release navigating back to their tree of origin and up to their most recent pipe refugium in the tree, often climbing past lower pipes and natural refugia. While we did not monitor the boles during fires, they likely hosted a diverse assemblage of vertebrates and invertebrates rapidly ascending to avoid the fire. The triggers for this vertical exodus are not well-understood, though chemoreception, sight, auditory cues, thermal sensing, or combinations of these mechanisms are likely at play (Grafe et al. 2002; Brennan et al. 2011; Dell et al. 2017).

The canopy layer of the landscape is often ignored by researchers in most systems, likely due to the technical difficulties involved. This is problematic as canopies represent significant portions of the habitat for many species, can be biodiversity hotspots, and contribute to the biogeochemical process (Rinker et al. 2001; Lowman 2009; Nakamura et al. 2017). In addition to treefrogs, we incidentally captured anoles, scorpions, spiders, wasps, cockroaches, and other insects, often in large quantities. Habitat for many of these animals, along with epiphytic plants, fungi, and bacteria, includes all three dimensions of the landscape and we should not assume that the dynamics observed on the ground are the same at the top of a tree (Nakamura et al. 2017, and those cited within). In an open canopy, where each mature tree is isolated from other trees, these differences may even be exaggerated. Factors such as weather effects, prey availability, and predation risk could have more extreme trade-offs given the isolation. The pine-dominated ecosystems of the southeastern US do not have the closed canopies of other forests; however, we believe that this patchiness of the canopy layer provides additional research questions to explore.

## Conclusions

Pinewoods treefrogs climb tall pines to escape fire but descend when pre-fire ground conditions return. We observed no major difference in survival among treatments for this specialist species, but frogs in trees in burned areas moved less within the tree. We recommend future efforts examine upland dynamics and utilize before-after-control-impact experimental designs when investigating prescribed fire effects (Pilliod et al. 2003). We also suggest future researchers consider supplementing the traditional ground and breeding pond approach to studying treefrogs with techniques like those presented here that can push into the vertical element of their habitat. This work is a significant step in elucidating fire effects on an often-overlooked group of amphibians. Furthermore, this project increases the dimensionality of our knowledge of hylid population dynamics.

## Data Availability

The datasets created and analyzed during the study are available at 10.2737/RDS-2022-0072.

## Abbreviations

COVID-19: Coronavirus disease 2019
cm: Centimeter
°C: Degree Celsius
DWP: Disney Wilderness Preserve
*D.*: *Dryophytes* (genus)
e.g.: Exempli gratia — for example
Fig.: Figure
FL: Florida
ha: Hectare
in: Inches
i.e.: id est — that is
m: Meter
mm: Millimeter
NEON: National Ecological Observatory Network
PVC: Polyvinyl chloride
psi: Pounds per square inch
R: R statistical software
RAD: Rapid ascension descension
SRT: Single rope technique
SVL: Snout-vent length
TNC: The Nature Conservancy
TSF: Time since fire
US: United States
VIE: Visible implant elastomer
WAIC: Widely applicable information criterion
